# Role of Robotics and Artificial Intelligence in Oral Health and Preventive Dentistry – Knowledge, Perception and Attitude of Dentists

**DOI:** 10.3290/j.ohpd.b1693873

**Published:** 2021-07-15

**Authors:** Hoda Lotfy Abouzeid, Saurabh Chaturvedi, Khalid M. Abdelaziz, Fawziah Ahmed Alzahrani, Abdulhkeem Ali Salim AlQarni, Nasser M. Alqahtani

**Affiliations:** a Lecturer, Department of Prosthetic Dentistry, College of Dentistry, King Khalid University, Abha, Saudi Arabia. Conceptualisation, methodology, validation, performed examinations, data curation, wrote the manuscript.; b Assistant Professor, Department of Prosthetic Dentistry, College of Dentistry, King Khalid University, Abha, Saudi Arabia. Methodology, questionnaire validation, performed examinations, wrote the manuscript.; c Associate Professor, Department of Restorative Dental Sciences, College of Dentistry, King Khalid University, Abha, Saudi Arabia. Questionnaire validation, wrote the manuscript.; d General Dental Practitioner, Ministry of Health, Asir Province, Saudi Arabia. Questionnaire validation, performed examinations, data curation, wrote the manuscript.; e General Dental Practitioner, Daweni Medical Clinics, Abha, Saudi Arabia. Questionnaire validation, performed examinations, data curation, wrote the manuscript.; f Assistant Professor, Department of Prosthetic Dentistry, College of Dentistry, King Khalid University, Abha, Saudi Arabia. Questionnaire validation, performed examinations, wrote the manuscript.

**Keywords:** artificial intelligence, digital dentistry, oral health, questionnaire, robotics, survey

## Abstract

**Purpose:**

To assess the knowledge, attitude and perception of dentists (dental students, dental school graduates/interns, postgraduate dentists) of the role of robotics (R) and artificial intelligence (AI) in oral health and preventive dentistry. The null hypothesis was that dentists would not be aware of R and AI use in dentistry and would not be ready to accept them in oral health and preventive dentistry for dental care management and training.

**Materials and Methods:**

This was an observational cross-sectional study in which data was collected from a representative population in Saudi Arabia. 570 participants answered 26 closed-ended questions. The questionnaire’s validity and reliability were evaluated for vetting and remarks. The questionnaire collected demographic data of participants and their knowledge, perception and attitude about R and AI. Questions were to be answered with ‘yes’, ‘no’ and ‘I don’t know’. Descriptive statistical analysis was performed using the control chart technique and the chi-squared test, with statistical significance set at p < 0.05.

**Results:**

The majority of the participants (n = 313; 54.6%) were males. Dental students, dentist school graduates/interns, and postgraduate dentists comprised of 58.8%, 18.2%, and 23.0% respectively. Most of the respondents gave affirmative answers for knowledge, attitude and perception of R and AI (58.3%, 67.4%, and 60.3%, respectively). Participants agreed that R and AI is beneficial in dentistry and would provide better results. Most (83.3%) would be willing to be treated using R/AI and would recommend (84.5%) treatment with R/AI, as shown in the control chart by affirmative answers. These were significantly above the overall affirmative answers, as the corresponding point lies above 95% UCL (upper confidence limit).

**Conclusion:**

Most dentists were unacquainted with R and AI. Dentists had a positive attitude towards R/AI, but due to inadequate knowledge and understanding, its use and applications were very limited. There is significant need in the near future to increase awareness of this concept, as it may increase treatment efficiency and effectiveness.

With the current rapid advancements in technology, robotics (R) and artificial intelligence (AI) have become an indispensable part of our daily life. Robotics is concerned with the connection of perception to action; in this, artificial intelligence must have a central role if the connection is to be intelligent. Thus, these two are complimentary to each other but serve very different purposes. Robotics is a branch of technology that deals with designing, construction, operation and application of robots, while AI is a branch of computer science which involves developing computer programs to complete tasks that would otherwise require human intelligence. AI algorithms can tackle learning, perception, problem-solving, language-understanding and/or logical reasoning.^6,23^

Medicine and dentistry are not isolated from the effects of R/AI. In oral health and preventive dentistry, the application of R/AI is increasing at a high rate. Robots have been applied to induce oral analgesia, desensitise teeth, manipulate tissue to re-align and straighten irregular dentition, and improve the longevity of teeth.^9,11,12,15,20^ Further, it is possible that robots may be used to do preventive, restorative, and curative procedures in the future. Using characterisation tools, a variety of oral diseases are understood at the molecular and cellular levels and thereby prevented.^2,13, 25,28,29,31^ Dental robots have been used in teaching dental students, endodontic work (endo micro-robots), archwire bending and dental implantology.^12,25^

AI tools are basically limited to examinations and their interpretation, for example, radiography, cone beam computed tomography (CBCT), magnetic resonance imaging (MRI), differentiation between vital and pathological signs, employing deep learning methods such as convolutional neural networks (CNNs) which are effective at performing narrow classification tasks where large training datasets are available.^6,10^

Although evidence is lacking to date to support routine use of R and especially AI algorithms in real-world dental clinical practice, it is expected that with increasing academic and industrial interest, validated use of R and AI tools in dentistry should emerge rapidly, as in radiology and medicine.^29^

All over the world, the use of novel digital methods in oral health and preventive dentistry are evolving, and their acceptance is increasing exponentially.^29^ There is a need for dental students, dental school graduates and senior clinicians to develop the necessary skills to handle advanced digital dental tools, so that they can implement R and AI in the dental field with greater efficacy.

The traditional dental curriculum contains very limited information about R and AI. However, the actual prevalence of such training and the level of R/AI knowledge amongst dental students, dental school graduates and clinicians remains unclear, despite of previous endeavours to quantify dental students’ perceptions of the use of R/AI technology in dental education. In 2015, Razavi et al^26^ proposed a haptics-based tooth drilling simulator for dental education. In 2017, Abe et al^1^ assessed attitudes of dental students towards the use of a full-body patient simulation system (SIMROID) compared to the traditional mannequin (CLINSIM) and found that use of SIMROID was an effective method in improving the attitude of students towards patients.^11^

Furthermore, to effectively engage dentists on this topic, it would be useful to understand their perception towards R/AI as a cohort. It is not unreasonable to assume that the current environment of enthusiasm towards R/AI exerts a great influence on dentists’ attitude and behaviour. Thus, it is timely to clearly identify the knowledge, attitude and perception of dentists towards R/AI. Social-psychological theory describes that attitudes have at least two interdependent components: cognitive perceptions (the way the facts are understood) and affective emotions (the way one feels about the facts).^7^ Attitudes should be integrated as an anecdotal component when considering clinical decision making among general practitioners.^18^ Supplementary studies have shown an association between attitudes and clinical behaviour.

Various studies have been conducted around the world in the medical^4,6,22^ and dental fields^2,4,9,15,29^ about R and AI, with surveys conducted in radiology^10^ and medicine,^24^ for instance, but to the best of the present authors’ knowledge, no survey has been conducted until now in field of oral health and preventive dentistry. Little is known related to the attitudes and acceptance of R/AI among dentists. Additionally, there are no studies related to R/AI in Saudi Arabia. The present authors found many reviews on R/AI,^5,20,28^ on various index platforms, but studies on R/AI from the clinician’s point of view were not reported in the dental field. Thus, the research question arose as to whether dentists in Saudi Arabia had knowledge about the use of R/AI in dentistry. Thus, the present study aimed to assess the knowledge (based on their level of education), attitude and perception of dentists (dental students, dental school graduates/interns, postgraduate dentists) towards R/AI in Saudi Arabia. The null hypothesis formulated was that dentists would not be aware of R/AI use in dentistry and would not be ready to accept its role in oral health and preventive dentistry for dental care management and training.

## Materials and Methods

### Type of Study and Sampling

This was an observational, cross-sectional study, in which data were collected from a representative population in Saudi Arabia (dental students involving participants from the 1st year of dental school study up to the final year, dental school graduates/interns group involving participants who completed dental school studies and practice dentistry independently, and/or interns who completed their final year in dental school and required a compulsory internship to become a licensed dentist, postgraduate dentists involving participants who were persuing postgraduate and specialty training during the 7 months from 1 May to 30 November 2019). A quota sampling (nonprobability sampling) technique was used. A total of 750 individuals participated in the study.

### Ethical Considerations

This study was conducted in compliance with the Declaration of Helsinki; ethical approval (number: src/eth/2018-19/100) was given by the ethics committee of the King Khalid University College Of Dentistry, ABHA, KSA. The participants provided their informed consent. Participation was voluntary and there were no incentives. Data protection and anonymity were guaranteed.

### Questionnaire

Questions related to general information about R/AI, its role and application in oral health and preventive dentistry were included. These questionnaires were distributed personally to the participants within reachable areas, and otherwise distributed online as google forms throughout the country, using different platforms for communication, e.g. WhatsApp, e-mail. The questionnaires were also distributed at a conference entitled “Excellence in Education Innovation and Patient Care”, held in Jeddah (KSA) on 21–24 October, 2019, and attended by dental professionals from all over the country. Each participant’s communication data was collected and coded. Twice every week, all participants were reminded to return the questionnaire with their responses. Wherever essential, detailed conversation was offered to the respondents and clarification was provided regarding the study and its use.

A unique custom-designed questionnaire was used and was influenced by a few previous studies in the medical field^2,10,24^ (Appendix 1). The questionnaire contained 26 closed-ended questions. Its validity and reliability were evaluated by 4 prosthodontists and a psychometrician for vetting and remarks. The recommended modifications were implemented to ensure its cogency. Also, the questionnaire was validated in a pilot study on 105 participants. After analysing the dataset of the pilot study, consistent responses were noticed, which depicted high internal consistency for the questionnaire, with an overall Cronbach’s alpha of 0.82. The Cronbach’s alpha for knowledge, perception and attitude was 0.84, 0.80, and 0.83, respectively. The questionnaire used in the study consisted of two parts. The first part included the dentists’ demographic data (gender, qualification, years of experience in dentistry); the second part consisted of questions on R/AI. In the areas of knowledge (questions 1–7), perception (questions 8–16) and attitude (questions 17–26), the respondents were required to answer with “yes”, “no” and “I don’t know”.

### Statistical Analysis

In order to achieve the outlined objectives, the scores were calculated based on the responses given by participants. A single investigator analysed all returned questionnaires. A database was constructed using Microsoft Excel (Microsoft; Redmond, WA, USA) and imported into SPSS version 20 (Chicago, IL, USA) for statistical analysis. Descriptive statistical analysis, which included frequency and percentages, was used to characterise the data and report the diversity of the sample employed in this research. Association with the factors was tested for statistical significance using the chi-squared test, with significance set at p < 0.05. Answers with unusually high proportions and relatively higher knowledge levels were evaluated by the control chart technique.

## Results

The survey was conducted among 750 participants; 628 completed the whole survey, yielding a response rate of 83.7%. Fifty-eight of them were excluded from final analysis based on a negative answer to the first ‘knowledge’ question (‘Have you heard about artificial intelligence and robotics in dentistry?’). Thus, the final analysis was done on 570 participants.

### Demographic Characteristics

Among the final study participants, a majority were males (n = 313; 54.6%). Dental students, dental school graduates/interns, postgraduate dentists comprised 58.8%, 18.2%, and 23.0%, respectively (Table 1).

**Table 1:** Demographic data of participants

Demographic variable		No. (N = 570)	%
Gender	Male	313	54.9%
	Female	257	45.1%
Qualification	Dental students	335	58.8%
	Dental school graduates/interns	104	18.2%
	Postgraduate dentists	131	23.0%
Years of experience in dentistry	1–5 years	432	75.8%
	5–10 years	71	12.4%
	>10 years	67	11.8%

### Knowledge, Perception and Attitude about R/AI

#### Knowledge about R/AI

Of the total 628 participants, 570 (90.7%) had heard of robotics and AI in dentistry, but only 40 (7%) were aware of the difference between robotics and artificial intelligence.

A total of five statements (Q3–Q7) regarding knowledge on robotics and AI were assessed to judge the knowledge level of respondents. Respondents gave statistically significantly more affirmative answers on the four items Q3, Q4, Q5 and Q6 (p < 0.0001), but the last statement, Q7 (Is AI used in detection of oral cancer in its early stages, such as during health campaigns?), received equal numbers of ‘yes’ and ‘no/don’t know’ answers (p = 0.753) (Table 2).

**Table 2:** Knowledge about robot technology

Statement about robot technology (N = 570)	Yes		No		Don’t know		Affirmed vs not affirmed	
	n	%	n	%	n	%	chi- squared	p-value
Q3: Robot technology is used to assist with patient diagnosis and the development of an integrated treatment plan.	345	60.6%	96	16.7%	129	22.7%	25.26	<0.0001
Q4: Robots are used in measurement of vital signs such as pulse, breathing, temperature, blood pressure, and ECG	346	60.6%	83	14.6%	141	24.8%	26.11	<0.0001
Q5: Artificial intelligence is used for examinations and their interpretation, e.g. radiographs, CBCT, MRI, differentiation between vital and pathological signs.	337	59.1%	97	17.0%	136	23.9%	18.98	<0.0001
Q6: Artificial intelligence is used in pathology, accurate reading of tissue samples, diagnosis.	337	59.1%	107	18.8%	126	22.1%	18.98	<0.0001
Q7: Artificial intelligence is used in detection of oral cancer in its early stages, such as during health campaigns.	294	51.6%	101	17.6%	175	30.7%	0.57	0.7530
Overall	58.3% (54.3% – 62.3%)		16.9% (13.8% – 20.0%)		24.8% (21.3% – 28.3%)			

**Fig 1:**
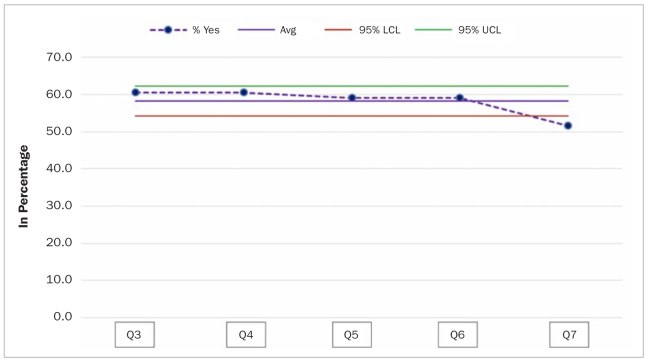
Control chart showing answers to Q3–7 with lower and upper confidence limits.

Further, the control charts showed that the rate of affirmative answers on Q7 (51.6%) was statistically significantly lower than for the overall affirmative answers (Q3 and Q4: 60.6%), as the corresponding point lies below the 95% LCL (lower confidence limit) (Fig 1).

#### Perception about R/AI

A total of 9 questions/statements (Q8–Q16) regarding perception on R/AI were presented to respondents. Respondents gave statistically significantly more affirmative answers to all the statements except Q11 and Q13 (p > 0.05) (Table 3).

**Table 3:** Perceptions about robot technology

Statement (N = 570)	Yes		No		Don’t know/very little		Affirmed vs not affirmed	
	n	%	n	%	n	%	chi squared	p-value
Q8: R/AI Use in oral health and preventive dentistry is beneficial	484	85.0%	29	5.0%	57	10.0%	277.90	<0.0001
Q9: Automated surgical robot in the area of maxillofactial surgery is that which supports the surgeon in performing a certain operation or may act as a surgeon’s assistant.	347	60.9%	106	18.5%	117	20.6%	26.98	<0.0001
Q10: In the field of orthodontics, artificial intelligence can provide a more accurate digital view of the mouth than the traditional method, and predict the movement of teeth and the final treatment of teeth and work applications with wire, as opposed to the laboratory.	393	69.0%	70	12.2%	107	18.8%	81.85	<0.0001
Q11: In endodontic treatment, working robots may reduce possible treatment errors and increase the quality of treatment.	294	51.6%	126	22.1%	150	26.3%	0.57	0.7530
Q12: R/AI may contribute to predicting the correct place in cases of dental implants through a 3D view before and during the process through an integrated simulation system.	363	63.6%	85	14.9%	122	21.5%	42.70	<0.0001
Q13: R/AI facilitates CAD/CAM and process of fabricating complete dentures	313	54.9%	93	16.4%	164	28.7%	5.50	0.0640
Q14: Can AI replace the dentist permanently?	200	35.2%	230	40.3%	140	24.5%	50.70	<0.0001
Q15: AI facilitates the storage of patient information, data and accessibility to it, quickly and accurately.	368	64.5%	75	13.1%	127	22.4%	48.34	<0.0001
Q16: Can robots contribute to increased career productivity, medical education, and awareness in the community and individuals?	330	57.9%	90	15.8%	150	26.3%	14.21	0.0008
Overall	60.3% (56.3% – 64.3%)		17.6% (14.5% – 20.7%)		22.1% (18.7% – 25.5%)			

**Fig 2:**
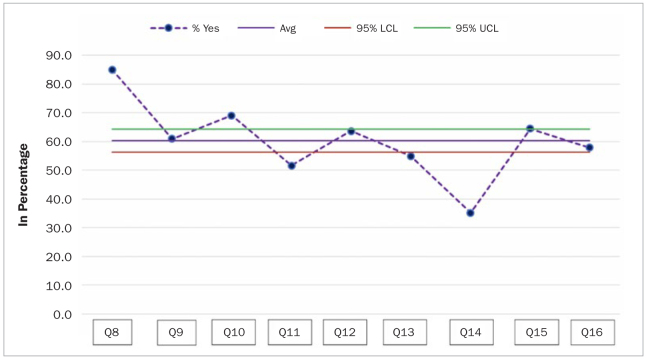
Control chart showing answers to Q8–16 with lower and upper confidence limits.

Upon examination of the control charts, it became evident that affirmative answers to Q8 (85.0%) and Q10 (69.0%) statements were statistically significantly more frequent than for the overall affirmative answers, as the corresponding point lay above the 95% UCL (upper confidence limit). In contrast, the affirmative answers to Q11, Q13 and Q14 were statistically significantly below the overall affirmative answers, as the corresponding point lay below the 95% LCL (Fig 2).

#### Attitude towards R/AI

Out of 10 questions/statements (Q17–Q26) regarding attitudes R/AI, statistically significantly more affirmative answers were given to all the statements except Q20 and Q21 (Table 4).

**Table 4:** Attitude towards robot technology

Attitude items (N = 570)	Yes		No		Neutral/DK		Affirmed vs Not Affirmed	
							chi sq	p-value
Q17: Would you recommend treatment with R/AI?	475	83.3%	95	16.7%	0	0.0%	253.3	<0.0001
Q18: Would you prefer treatment done with R/AI on yourself, if needed?	482	84.5%	88	15.5%	0	0.0%	272.3	<0.0001
Q19: Would you prefer to work in the robot simulation lab for training in root canal, crowns, bridges and fillings etc.?	347	60.9%	223	39.1%	0	0.0%	27	<0.0001
Q20: Would you prefer to receive lectures or workshops from a robot?	290	50.7%	173	30.4%	107	18.8%	0.175	0.916
Q21: Would receiving information from a teaching robot increase self-confidence more than in traditional classroom?	274	48.1%	162	28.4%	134	23.6%	0.849	0.654
Q22: If you had the opportunity to work in a team that includes a robot as a participant, would you agree to join?	371	65.1%	87	15.2%	112	19.7%	51.9	<0.0001
Q23: Would you like to learn about R/AI in future?	500	87.7%	22	3.8%	48	8.5%	324.4	<0.0001
Q24: Has the time come for students, doctors and individuals working in the university to accept R/AI techniques?	371	65.1%	75	13.1%	124	21.8%	51.9	<0.0001
Q25: Do you think the application of R/AI will enhance your clinical practice?	363	63.7%	107	18.8%	100	17.5%	42.7	<0.0001
Q26: Is there a need to switch to a secure digital environment using artificial intelligence applications and create a healthy system with all the latest technologies?	367	64.3%	70	12.3%	133	23.4%	47.18	<0.0001
Overall	67.4% (63.6% – 71.2%)		19.3% (16.1% – 22.5%)		13.3% (10.5% – 16.1%)			

**Fig 3:**
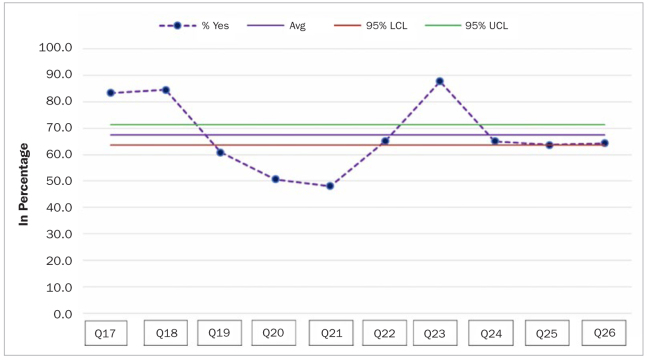
Control chart showing answers to Q17–26 with lower and upper confidence limits.

The control charts showed that affirmative answers to Q17 (83.3%), Q18 (84.5%) and Q23 (87.7%) were statistically significantly more frequent than for the overall affirmative answers, as the corresponding point lay above the 95% UCL (upper confidence limit). In contrast, affirmative answers to Q19, Q20 and Q21 were statistically significantly less frequent than for the overall affirmative answers, as the corresponding point lay below the 95% LCL (Fig 3).

## Discussion

It is a well-known fact that the medical world is highly influenced by R/AI. R/AI is increasingly influencing the field of dentistry as well, as proven by the mounting number of studies^1,9,11,12,15,20,26^ on R/AI applications dentistry. Given this continuous change, it is necessary for students and clinicians to update their knowledge in field of R/AI. This study was conducted to assess the status of knowledge, perception and attitudes about R/AI among dental students and dentists in Saudi Arabia. The results of the study showed that R/AI will have a profound future impact on dental practice. This was also acknowledged by the majority of respondents in our cohort, with 85% agreeing that R/AI is useful for oral health and preventive dentistry and it would play an important role in dental care management and training (Q.8). Thus, the null hypothesis was rejected, proving that dentists/dental students were aware of R/AI use in dentistry.

Although most of the participants agreed (40.3%) that R/AI will not replace dentists completely (Q.14), similar to studies in radiology and other medical fields,^10,24,29^ almost 35.2% agreed that this would happen in the future and 24.5% were not sure. This could be a signal to all dentists that the impact of R/AI in dentistry cannot be overlooked and that there is an urgent need to enhance the knowledge and training in R/AI.

Basically, AI addresses the crucial questions of what knowledge is required in any aspect of thinking, and how that knowledge should be represented and used, whereas robotics challenges AI by forcing it to deal with real objects in the real world.^20,24,25,29^ In our opinion, the problem lies in the basic curriculum of the dentistry: during the entire BDS course, a graduate is currently not exposed to the advanced R/AI technological progress in dentistry. Even if a few topics are discussed, the specific importance is not stressed at any time, and the field remained unexplored. This was gleaned from the responses to questions (Q2) about differences between R and AI (78% ‘no’ answers). Mostly, students work on patient simulators commonly known as ‘phantom heads’ or are given demonstrations about implant treatment with planning software only. If not for this, knowledge of R/AI would be equivalent to nil. Students are not exposed to R/AI, and even after graduation, continuing training (e.g. effective workshops) to enhance knowledge is essentially not available. This was shown by the response to questions about knowledge of R/AI applications in different aspects of dentistry; approximately 40% of respondents answered ‘no’ or ‘I don’t know’ (Q 3–7).

Almost all branches of dentistry are positively affected by R/AI, especially surgery, prosthodontics, restorative dentistry, implantology and diagnosis, radiology, treatment planning, oral pathology, and orthodontics, as has been well documented in previous reviews.^9,18,20,25,28,29^ In our study, the participants agreed with this, as shown by their responses to questions on perception of R/AI. Perception represents an individual’s own view or the interpretation of something. Around 60% of the participants were of the view that R/AI could work efficiently and assist dentists in different fields of dentistry (Q9–16). The use of automated robots in surgery, orthodontics, and implants was affirmed by the respondents. A surgical robot system has been used to program robots to perform the surgical procedures such as milling of bone surfaces, drilling holes, deep saw osteotomy cuts, and orthognathic surgery, etc. A surgeon can activate the task needed and the robot performs the pre-programmed tasks.^5,25^

Similarly, micro endodontic robots could provide safe, accurate, and reliable root canal treatment for patients. This would reduce the dependency on the skills of the dentist and minimise human error.^25^ Nano diagnostic devices could be used for early disease identification at the cellular and molecular levels. Dental nanorobots might use specific motility mechanisms to penetrate human tissue with navigational precision, acquire energy, and sense and manipulate their surroundings in real time.^20,28^ The overall perception of the cohort in this study was good towards R/AI. This could be a positive sign towards incorporation of R/AI in clinical practice, and also indicated that with the proper use of the technology, dentistry would progress, and better treatment could be provided to the patients.

Various clinical situations in routine dentistry could be performed better with the use of technology. This, however, requires that the clinician have a positive attitude towards the technology. In the present study, similar to previous studies^2,10,24,29^ in radiology, surgery, and ophthalmology, participants had positive attitudes toward R/AI application in oral health and preventive dentistry. 83% of our participants recommended and even showed willingness to be treated by robots, if needed (Q17,18). 60.9% of the participants were enthusiastic about working and being trained in the robot simulation lab for root canal treatment, crowns, bridges and fillings, etc (Q19). The use of R/AI as a teaching tool is also increasing exponentially. The increased interest of a majority of participants to learn things by R/AI was very clearly conveyed in the affirmative answers about receiving information, lectures or workshops (50.7%), working in a team that includes a robot as a participant (65.1%) and above all, having robot as a teacher would increase the self-confidence (48.1%) (Q20-22). This requires special attention, because in order to increase the role of robotics in education, it is important to develop a well-defined curriculum. Curricula, learning materials, and teachers’ training should be developed and individualised for each type of robotic technology and level of dental training.

Almost the entire cohort (93%) (Q23) was willing to attend a course related to R/AI and want to learn about it in detail. Learning is a curve which will never flatten, as illustrated by the results of our study: almost all participating students or even senior dentists were ready to enhance their knowledge.

Nevertheless, if the dentist has a positive attitude towards and a favorable perception of R/AI, then there would always be demand for better understanding of any technology and its application. More than 60% of the participants answered ‘yes’ about application of R/AI in enhancing clinical practice (Q24-26), accepted R/AI techniques at the university, and affirmed the need to switch to a secure digital environment using artificial intelligence applications and create a health-care system with all the latest technologies.

One important fact is that the digitalisation of dentistry, especially incorporation of R/AI, has led to a technical revolution, but it comes with associated risks and ethical challenges. Also, it is well known that R/AI – like any technology – will only be successful in the long term if it enjoys a high and sustained level of social acceptance. Ethical issues are related to data management: storage, sharing, and use (the latter also including data manipulation).^19,30,33^

With increased use of R/AI, the dental practitioner-patient relationship would be impacted, as it is traditionally a direct two-way relationship which would become more indirect as a result of the integration of technical systems into patient treatment.^16^ Digital literacy for dentists will become mandatory, as problems will arise if the dental practitioner has inadequate mastery of a new technology. Defining responsibility will be difficult with the new, complex technical systems, as many people are involved in the development, operation, and application of the technology.^12,17^ A practitioner’s self-image and professional expertise would be affected, as well as public perception, and hand manual skills might be questioned. A major issue would be the cost of treatment, which is also referred to as the ‘amortization trap’, associated with increased risk of overdiagnosis and overtreatment, meaning that a medical indication for the use of a technological aid may be overused. Finally, the lack of clinical and scientific evidence would result in low confidence among dentists and patients.^12^

### Recommendations

Based on the results of this study, it is recommended that R/AI be introduced into the undergraduate curriculum, so that students receive basic understanding these technologies. Furthermore, continuing dental education programs should be conducted frequently to enhance dentists’ knowledge of R/AI, and dental professionals should be informed and motivated towards its implementation to provide improved treatment in indicated cases.

### Limitations

The limitations of the study included its limited sample size (628) and low response rate (83.7%) relative to the area covered. Thus, the findings should not be generalised. Inherent limitations of cross-sectional studies (non-response from the participants^23^), closed-ended questionnaire (suggestions or ideas given to participants, simplistic responses to complex questions, misinterpretation of questions may go unnoticed^24,25^) and nonprobability sampling technique (depends heavily on the expertise of the researchers) should also be taken into account.

Future studies are recommended, for instance, in collaboration with government agencies to cover the entire Kingdom of Saudi Arabia. This would provide a more complete scenario regarding R/AI among working dentists. Also, our cohort involved dentists/students of different nationalities, so a study segregating nationalities is recommended to develop the policies appropriate to a given country.

## Conclusion

Most dentists are still unacquainted with R/AI. In general, dentists had positive attitude, but due to inadequate knowledge and understanding of R/AI, its use and application was absent or very limited. There is significant need in the near future to increase awareness of this concept, as it may increase treatment efficiency and effectiveness. This could be achieved by inclusion of R/AI in the undergraduate curriculum in a coordinated manner and by continuing dental education programs.

## Appendix 1

### Questionnaire: Knowledge, Perception and Attitude of dentists towards artificial intelligence and robotics in dentistry

#### Demographic Data

**Gender:**

Male

Female

**Qualification:**

Dental Student

Dental school graduate/intern

Post-graduate dentist

**Years of experience in dentistry**

1–5 years

5–10 years

>10 years

#### Questions Related to Knowledge

1. Have you heard about artificial intelligence and robotics in dentistry?

Yes

No

(If the answer is ‘no’, do not proceed with the questionnaire)

2. Do you know the difference between artificial intelligence and robotics?

Yes

No

Very little

Q. Mark your answer to the following statements:

**Table:**

Questions/Statement	Yes	No	I don’t know
3. Robot technology is used to assist with patient diagnosis and the development of an integrated treatment plan.			
4. Robot are used in measurement of vital signs such as pulse, breathing, temperature, blood pressure and ECG			
5. Artificial intelligence is used in examinations and their interpretation, e.g. radiographs, CBCT, MRI, differentiation between vital and pathological signs.			
6. Artificial intelligence is used in the field of pathology for accurate reading of tissue samples and assists in the diagnosis.			
7. Artificial intelligence is used in detection of oral cancer in its early stages, such as during health campaigns.			

#### Questions Related to Perception

8. Is R/AI use in dentistry beneficial?

Yes

No

Don’t know

Q. Give your opinion on the following statements:

**Table:**

Questions/Statement	Yes	No	I don’t know
9. Automated surgical robots in oral and maxillofacial surgery work with the surgeon to perform a certain operation or may act as a surgeon’s assistant.			
10. In the field of orthodontics, artificial intelligence can provide a more accurate digital view of the mouth than the traditional method, and predict the movement and the treatment of teeth, and work applications with wire rather than the laboratory.			
11. In endodontic treatment, working robots may reduce possible treatment errors and increase the quality of treatment.			
12. R/AI may contribute to predicting the correct placement of dental implants, providing 3D views before and during the process through an integrated simulation system.			
13. R/AI facilitate CAD/CAM and process of teeth arrangement			
14. Can artificial intelligence replace dentists permanently?			
15. Artificial intelligence facilitates the preservation of patient information, data and accessibility quickly and accurately.			
16. Can robots contribute to increased career production, medical education and awareness in the community and individuals?			

#### Questions Related to Attitude

17. Would you recommend treatment done with R/AI?

Yes

No

18. Would you prefer treatment with R/AI done on yourself, if needed?

Yes

No

19. Would you prefer to work in the robot simulation lab for training in endodontics, crowns, bridges and fillings etc.?

Yes

No

20. Would you prefer to receive lectures or workshops from a robot?

Yes

No

Neutral

21. In your opinion, does receiving information from a teaching robot increase self-confidence more than in a traditional classroom?

Yes

No

Neutral

22. If you had the opportunity to work in a team that included a robot as a participant, would you agree to join?

Yes

No

Neutral

23. Would you like to learn about R/AI in the future?

Yes

No

Neutral

24. Has the time come for students, doctors and individuals working in the university to accept R/AI techniques?

Yes

No

I don’t know

25. Do you feel that application of R/AI could enhance your clinical practice?

Yes

No

I don’t know

26. Do you think that there is a need to switch to a secure digital environment using artificial intelligence applications and create a health-care system with all the latest technologies?

Yes

No

I don’t know
